# Case of Sudden Death from (Supposed) Heart-Clot

**Published:** 1853-06

**Authors:** 


### *Case of sudden death from (supposed) Heart-clot.*

*My Dear Doctor,*—You will receive in a few days, the specimen of fibrinous concretion of the heart, which I promised to you when in Buffalo last.

Some of its more interesting features are obliterated by the action of alcohol. One or two points are worthy of notice, viz: the slight degree of endocarditis, and the fragile manner in which the clots were attached to the walls of the heart. I have not been able to demonstrate the existence of blood-vessels within the clots; though I think you will agree with me that they have the appearance of organized bodies. In a case of collapse of lung which I inspected, post-mortem, a year ago, there were concretions like these, but softer, less striated, more yellow, and entirely free from attachment to the ventricular parietes. The history of this specimen is briefly this:

*June 4th*, 1852. I was called to John Connor. When I arrived there was no action of the heart, but he gasped once or twice. I could not ascertain the previous history of the case, farther than that he came a few days before to the house where he died, and had been engaged in farm labor up to the day of his death. He had a presentiment of death, and spent much of his time in prayer. Had been subject for some time to palpitation and occasional syncope.

I held a post-mortem eight hours after death. On opening the thorax the lungs were found tubercular to a considerable extent, with firm pleuritic adhesions. The heart, externally, presented nothing unusual. Internally, a large fibrinous concretion was found on the right side of the heart, with a pyramidal base filling the apex of the ventricle, and extending through the auricle into the vena cava descendens. On the left side a smaller clot, less firmly organized, extended from the ventricle through the auricle into the pulmonary vein. These clots, as preserved in the specimen, are about six inches in length. They are not entire, having been cut off with the great vessels. The larger clot, at its base, is more than an inch in diameter.

A small granular liver, and a large "ague cake" in the spleen, were the only other organic changes.

The literature of heart clot, though scanty, is not uninteresting. I have somewhere seen a monograph by C. D. Meigs, which contains much that is noteworthy in this connection, in spite of a style turgid and bombastic; a sort of Carlylese run mad, in which the most impossible nouns are coupled "tail end to" with the most unheard-of adjectives, and verbs fresh from the mint of transcendentalism. (By the way, have you seen anything in Buffalo of a new disease, having verbal *softness* for its proximate cause, affecting chiefly the *parts of speech*, confining its ravages to writers and latter-day illuminati, and which may be called *Carlyleites?*) This monograph of Dr. Meigs details cases of women, who, having lost much blood during accouchment, fell into syncope, from imprudently assuming the erect position, during which syncope coagula formed in their hearts, producing sudden death.

But this matter of fibrinous clots is no new thing. Dr. Edward May, of the olden time, sent to Severinus an account of an eel which he took from a human heart. This account he designates "*Historia mirabilis anguis bifidi.*" This wonderful eel had a head and a tail, (the only organs actually necessary to make an eel,) the head biting the walls of the ventricle, and the tail disporting itself *ad libitum* among the venæ cavæ. This eel was the Sea Serpent of those days, occasioning much learned discourse. Severinus swallowed the eel, or rather the story, and taught his students that ulcers of the heart are not mortal—alluding to the biting of the eel.

Kerkingrius got himself into trouble with Malpigbi, Tulpius, and Pechlinus, on this very same question. He made the usual mistake of being in advance of his times, and declared these polypi, (as they were then called,) pseudo-polypi — mere clots of blood. Whereupon Pechlinus "pitched into him," telling Kerkringius that he did not know what a polypus was. He says in a style very much like that of some metropolitan physicians of the present day: "true polypi there certainly are, but these polypi of Kerkringius, are indeed pseudo-polypi, and every blind barber knows them abundantly well, *(tam est vulgaris et lippis tonsoribus notus.)* The shop boys make such polypi by pouring vitriolic acid into the veins."

Poor Kerkringius! He has slept with his fathers for many a generation, and now the tardy tribute of merited praise is first rendered to his genius by an obscure writer in a far distant land!

Furthermore, and lastly, so recent an authority as John Bell denies the existence of these concretions, asserting that coagulation not being a vital process, cannot take place in the heart — a position, it is needless to say, long since abandoned. S. B. H.

